# Sensitivity to Climate and Weather Changes in Euthymic Bipolar Subjects: Association With Suicide Attempts

**DOI:** 10.3389/fpsyt.2020.00095

**Published:** 2020-03-05

**Authors:** Marco Di Nicola, Marianna Mazza, Isabella Panaccione, Lorenzo Moccia, Giulia Giuseppin, Giuseppe Marano, Paolo Grandinetti, Giovanni Camardese, Domenico De Berardis, Maurizio Pompili, Luigi Janiri

**Affiliations:** ^1^ Department of Psychiatry, Fondazione Policlinico Universitario “A. Gemelli” IRCCS, Rome, Italy; ^2^ Institute of Psychiatry, Università Cattolica del Sacro Cuore, Rome, Italy; ^3^ Department of Mental Health, ASL Roma 1, Rome, Italy; ^4^ Department of Mental Health, AUSL Modena, Modena, Italy; ^5^ Department of Mental Health, Psychiatric Service of Diagnosis and Treatment, Hospital “G. Mazzini”, Teramo, Italy; ^6^ Suicide Prevention Centre, Department of Neurosciences, Mental Health and Sensory Organs, Sant’Andrea Hospital, Sapienza University of Rome, Rome, Italy

**Keywords:** weather, climate, meteoropathy, bipolar disorder, suicide, personalized medicine

## Abstract

**Background:**

Climate and weather are known to affect multiple areas of human life, including mental health. In bipolar disorder (BD), seasonality represents an environmental trigger for mood switches, and climatic variables may contribute to recurrences. Several studies reported seasonal and climatic-related variations in the rate of suicide attempts. Suicide risk is relevant in BD, with approximately 25% of patients attempting suicide. Therefore, this study aimed to assess sensitivity to weather and climatic variations in BD subjects and its relationship with lifetime suicide attempts.

**Methods:**

Three hundred fifty-two euthymic BD and 352 healthy control subjects, homogeneous with respect to socio-demographic characteristics, were enrolled. All participants were administered the METEO-Questionnaire (METEO-Q) to evaluate susceptibility to weather and climatic changes. We also investigated the potential relationship between sensitivity to climate and weather and lifetime suicide attempts in BD patients.

**Results:**

METEO-Q scores and the number of subjects reaching the cut-off for meteorosensitivity/meteoropathy were significantly higher in BD patients. Within the clinical group, BD subjects with lifetime suicide attempts obtained higher METEO-Q scores, with no differences between BD-I and BD-II. The number of suicide attempts directly correlated with METEO-Q scores. The presence of suicide attempts was associated with the physical and psychological symptoms related to weather variations.

**Discussion:**

Our findings support the relevance of sensitivity to weather and climate variations in a large sample of BD subjects and point out the association of this feature with lifetime suicide attempts.

## Introduction

The strict relationship between health and environment is well known, and there is increasing evidence that climate and weather affect multiple areas of human life, including mental health (1). For example, “pleasant weather” (e.g., elevated temperature or barometric pressure) seems to be related to higher mood levels, better memory, and broadened cognitive style (2), whereas high levels of humidity have been associated with impaired concentration and increased sleepiness (3). Though generally experienced only to a mild degree, some individuals display extreme sensitivity to weather and climatic changes (4, 5).

Psychiatric patients have been described to be particularly vulnerable to climate and weather changes in terms of clinical severity, admission rates, duration of hospitalization, recurrences, and outcome (6–9). Accordingly, a recent study found correlations between different weather conditions and the number of patients accessing the emergency room for psychiatric symptoms (10).

Among psychiatric diseases, bipolar disorder (BD) is the fifth highest as to self-reported disability, represents a significant public health problem, and is saddled with a markedly high mortality rate from suicide (11, 12).

There is a consensus on considering the changing of the seasons as one important environmental trigger for mood switches in BD, and emerging evidence suggests that climatic variables may contribute to recurrences (13, 14). Some studies reported a relationship between hospitalizations for mania and hours of sunshine, or temperature (15–17). Christensen and colleagues (18) found that several meteorological factors, including changes in minimum and maximum temperature, rainfall, atmospheric pressure, and cloudiness, might contribute to triggering new episodes in bipolar patients. Additional findings entailing climate variables include the observed association between hospital admissions for bipolar depression and temperature (19), as well as the prevalence of depressive episodes in relation to latitude (20), although other researchers reported different results (21). Interestingly, a recent study found a significant inverse association between the maximum monthly increase in solar insolation and the age of onset of type I BD (22).

Several evidences described seasonal and climatic-related variations in suicidality, with different patterns in men and women (23–28). Of note, climate and weather seem to independently influence suicidal behavior, both in psychiatric patients and in the general population. Fountoulakis et al. (29) described a correlation between the number of suicides and environmental temperature in different European countries, and climate changes appeared to influence suicidality even more than socio-economic factors. These observations have been further supported by a recent study that found a significant impact of temperature fluctuations on suicide rates in a large sample of subjects across the USA and Mexico (30). Seasonal effects have been reported to play a role in the likelihood of suicidal behaviors in affective disorder patients (31, 32). In manic BD subjects, higher suicide risk has been associated to rapidly increasing temperatures (33).

Following these considerations, we hypothesized that bipolar subjects would show a higher rate of sensitivity to climatic changes and that this feature could be related to suicide risk. Our group devised and validated a self-administered instrument, the METEO-Questionnaire (METEO-Q), to detect sensitivity to climate and weather changes in the general population and to assess their impact on symptom variations in psychiatric patients (34).

The aims of the study were: (i) to investigate sensitivity to weather and climate variation in euthymic bipolar subjects as compared to homogeneous healthy controls, using the METEO-Q; (ii) to evaluate the relationship of susceptibility to weather/climate changes with lifetime suicide attempts (SAs) in BD.

## Methods

### Participants

Participants were recruited among outpatients referring to the Day-Hospital of Psychiatry at the Fondazione Policlinico Universitario “A. Gemelli” IRCCS, Università Cattolica del Sacro Cuore, Rome, between January 2016 and May 2019. Inclusion criteria were: age 18 to 70; DSM-5 diagnosis of BD (type I and II); euthymia for at least three months; fluency in both spoken and written Italian. Exclusion criteria were: documented mental retardation or IQ < 70; alcohol/substance use disorders in the last three months; comorbidity with neurological or endocrine diseases. Psychiatric comorbidities were assessed by the Structured Clinical Interview for DSM-IV Axis I and II disorders (SCID-I/P; SCID-II) (35, 36). All included patients had been under stable pharmacological treatment for at least six months. The clinical sample finally consisted of 352 BDs (149 type I; 203 type II) subjects.

Healthy controls (HC) were recruited from community volunteers through advertisements. HC subjects were free of any psychiatric diagnoses as determined by the SCID-I, Nonpatient edition (SCID-I/NP) and the SCID-II (35, 37). Exclusion criteria were: history of brain trauma; lifetime neurological and endocrine illnesses; current psychopharmacological or psychotherapeutic treatment. None of them reported lifetime suicidal attempts. Three hundred fifty-two HC subjects were ultimately enrolled in the study.

Anonymity was guaranteed to all participants. The study protocol fully complied with the guidelines of the local Ethics Committee and was approved by the Institutional Review Boards in agreement with local requirements. It was conducted in accordance with Good Clinical Practice guidelines and the Declaration of Helsinki (1964) and subsequent revisions. All subjects enrolled gave their written informed consent before their inclusion in the study and participated without receiving any form of payment.

### Procedure and Assessment

A semistructured interview was employed to obtain demographic information, as well as medical/psychiatric history. SAs were assessed with a semistructured questionnaire including two questions: “Have you ever attempted suicide?”; “If yes, how many times have you attempted suicide?” SA was defined as “a self-inflicted, potentially injurious behavior with a nonfatal outcome for which there was evidence (either explicit or implicit) of intent to die” (38).

The assessment of mood state (euthymia for at least three months) was based on clinical and psychometric evaluations (i.e., Hamilton Depression Rating Scale < 8 (39); Young Mania Rating Scale < 6 (40).

All participants were administered the METEO-Questionnaire (METEO-Q) to evaluate susceptibility to weather and climatic changes. The device consists of 11 items and a structured checklist aimed at identifying the physical and psychological symptoms related to climate variations. Items and checklist symptoms are rated on a five-point Likert scale ranging from 0 (absent) to 4 (severe). The questionnaire also differentiates between “meteorosensitivity” and “meteoropathy”: subjects biologically susceptible to feel the effects of atmospherical events on mind and body are defined “meteorosensitive,” whereas individuals who develop a specific illness or a worsening of the existing diseases as a consequence of climatic changes are called “meteoropathic.” Different threshold values for detecting meteorosensitivity and meteoropathy in men and women were established (men: METEO-Q total scores >6 for meteosensitivity, > 9 for meteoropathy; women: METEO-Q total scores >8 for meteosensitivity, > 11 for meteoropathy) (34). Raters (MDN and MM) were specifically trained and showed good inter-rater reliability on all instruments (kN=0.80).

Upon evaluation, patients underwent a naturalistic maintenance treatment, with established mood stabilizers (lithium, valproate, carbamazepine) and atypical antipsychotics (olanzapine, quetiapine, aripiprazole, asenapine).

### Statistical Analysis

Statistical analyses were performed using the software IBM-SPSS Statistics, Version 24.0. Dichotomous data were described as numbers and percentages and compared using either the Chi-square test or Fisher’s exact test; continuous data were reported as mean (M) ± standard deviation (SD) and compared by Independent-Samples T-test or by non-parametric Mann–Whitney U test if not normally distributed. Bravais-Pearson or non-parametric Spearman’s rank correlation coefficients were reported as measures of association between continuous variables. Factors significantly associated with suicidal attempts in bivariate analyses were subjected to a multivariate logistic regression with SAs as dependent outcome measures. We examined all variables for multicollinearity. The Hosmer-Lemeshow goodness-of-fit statistic was used to check the fit of the model. Findings were reported as odds ratios (OR) and p values. All tests were two-tailed, with statistical significance set at p < .05.

## Results

### Demographic and Clinical Features in Bipolar vs. HC Subjects

All participants were Caucasians and homogeneous for age, gender, level of education, but not for marital status and employment condition (**Table 1**).

**Table 1:** Demographic and clinical data of euthymic bipolar disorder and healthy control subjects.

	Bipolar Disorders subjects	Healthy Controls	t/Z/χ2	p
N	352	352		
Gender (male) (n, %)	130 (37)	141 (40)	.817	.366
Age (M ± SD)	48.9 ± 11.4	48.8 ± 11.4	−1.67	.867
Educational level (years, M ± SD)	13.1 ± 3.2	13.3 ± 2.6	1.054	.292
*Marital status* (n, %)			17.08	**.001**
Single	118 (33.6)	114 (32.5)		
Married	161 (45.6)	203 (57.8)		
Separated/divorced	58 (16.5)	27 (7.6)		
Widowed	15 (4.3)	8 (2.1)		
*Employment condition* (n, %)			42.55	**< .001**
Student	13 (3.7)	8 (2.3)		
Occasionally employed	41 (11.6)	66 (18.8)		
Regular job	168 (47.7)	217 (61.5)		
Retired	41 (11.6)	34 (9.7)		
Unemployed	89 (25.3)	27 (7.8)		
*Clinical data*				
Current smoking (n, %)	177 (50.3)	149 (42.4)	4.081	.079
Onset of illness (age, M ± SD)	28.7 ± 11.9	–		
Duration of illness (years, M ± SD)	19.2 ± 12.1	–		
N° of affective episodes (M ± SD)	5.7 ± 5.1	–		
N° of hospitalizations (M ± SD)	2.1 ± 3.4	–		
Psychiatric comorbidity (n, %)	88 (25)	–		
Suicide attempts (n, %)	82 (23.3)	–		
N° of suicides (M ± SD)	1.54 ± .95	–		
*Bipolar Disorder diagnosis* (n, %)				
BD type I	149 (42.3)	–		
BD type II	203 (57.7)	–		
*Pharmacological treatment* (n, %)				
Lithium	162 (46)			
Antiepileptics mood stabilizers	208 (59)			
Atypical antipsychotics	218 (61.9)			
*Psychometric assessment* (M ± SD)				
HDRS	2.97 ± 1.3	2.78 ± 1.64	−1.213	.226
YMRS	1.21 ± .78	1.06 ± 1.42	−1.373	.170
METEO-Q Total	21.6 ± 11.8	13.4 ± 8.2	−9.666	**<.001**
METEO-Q Checklist	30 ± 18.6	16 ± 12.8	−10.475	**<.001**

Significant results are reported in **bold** characters.

BD, Bipolar Disorder; χ2, Chi-square test; HDRS, Hamilton Depression Rating Scale; M, mean; N, number of cases; p, statistical significance; SD, standard deviation; t, Independent Samples t-test; YMRS, Young Mania Rating Scale; Z, Mann-Whitney U test.

In BD subjects comorbid disorders were anxiety disorders (generalized anxiety disorder: n=18, 5.1%; panic disorder: n=14, 3.9%; social anxiety disorder: n=5, 1.4%), eating disorders (bulimia nervosa: n=6, 1.7%; anorexia nervosa: n=3, 0.8%), obsessive compulsive disorder (n=10, 2.8%); personality disorders (borderline: n=13, 3.7%; obsessive-compulsive: n=8, 2.3%; histrionic: n=5, 1.4%; avoidant: n=4, 1.1%; narcissistic: n=2, 0.6%).

METEO-Q Total and Checklist scores were higher in BD than in HC subjects (**Table 1**), with no differences between BD I and II types.

The rate of individuals reaching the cut-off for meteorosensitivity/meteoropathy was higher in bipolar subjects than in HC (meteorosensitivity: 5.4% vs. 16.3%; meteoropathy: 82.7% vs. 59.4%; χ^2^ = 39.42; p < .001), with no differences between BD I and BD II patients.

Women reported higher METEO-Q Total and Checklist scores both in the clinical sample (METEO-Q Total: 22.8 ± 11.4 vs. 19.3 ± 12.1; t=-2.517, p=.012. METEO-Q Checklist: 31.5 ± 19.1 vs. 27 ± 17.3; t=-2.013, p=.045) and in controls (METEO-Q Total: 15.2 ± 8.3 vs. 10.2 ± 6.8; t=-5.282, p < .001. METEO-Q Checklist: 18.2 ± 13 vs. 12.4 ± 11.7; t=-3.774, p < .001). Only in the HC group, a significant gender difference in reaching the cut-off of meteorosensitivity/meteoropathy was found, with higher rates in females (meteorosensitivity: 17.4% vs. 14.3%; meteoropathy: 63.5% vs. 52.4%; χ^2^ = 7.256; p=.027).

There were no correlations between METEO-Q scores (Total, Checklist) and HDRS and YMRS scores in both clinical and control groups.

### Sensitivity to Climate/Weather Changes and SAs in Bipolar Subjects

To fit our aims, we subdivided the clinical sample into bipolar subjects with (n=82) and without (n=270) a lifetime history of one or more SAs. The two groups did not significantly differ for socio-demographic and clinical features, except for age of onset (BD+SA vs. BD: 25.3 ± 10.3 vs. 30.9 ± 12.3; t=3.343, p=.001) and occupation typology, with higher rates of students and lower rates of regularly employed subjects in the BD+SA group (χ^2^ = 12.699, p=.013).

BD patients with lifetime SAs obtained significantly higher METEO-Q scores (METEO-Q Total: 24.8 ± 13 vs. 19.6 ± 10.3; t=-3.142, p=.002. METEO-Q Checklist: 37.6 ± 20.3 vs. 26.6 ± 16.2; t=-4.18, p < .001), with no differences between BD I and BD II subjects. The number of SAs directly correlated with METEO-Q Total (ρ=.217, p=.002) and Checklist (ρ=.334, p < .001) scores. No differences in the number of subjects reaching the cut-off scores for meteorosensitivity/meteoropathy were found in the two groups.

Finally, multivariate logistic regression analysis indicated that lifetime SAs were associated with the physical and psychological symptoms related to climate variations (METEO-Q Checklist: OR=1.03, p=.01).

## Discussion

BD is characterized by a high degree of psychopathological burden, with significant rates of relapses and recurrences. Many different factors, both individual and environment-related, have been recognized as triggers for the development of new episodes and the worsening of residual inter-episode symptoms. Among these factors, seasonality (41), the dysregulation of circadian rhythms (42), as well as abnormalities in the biological response to sunlight (43, 44) and moon cycle (45), have been demonstrated to play a significant role. Therefore, climate and weather sensitivity would be expected to be related to the course of the illness as well.

In this study, we found a higher susceptibility to weather and climatic changes in BD patients compared to homogeneous HC. Since patients were all euthymic and evaluated at different times of the year, results are likely not influenced by current mood state or specific seasonality, possibly representing an enduring trait (46).

The hypothesized neurobiological mechanisms influencing sensitivity to climate changes are complex and involve many factors, including different neurotransmission pathways, the immune and neuroendocrine systems, and the activity of genes regulating circadian rhythms (14, 47).

Recently, genetic variations in the circadian gene neuronal PAS domain protein 2 (NPAS2) have been proposed as a biomarker for a seasonal pattern in BD (41). Of note, both circadian rhythms and the response to sunlight and to climatic variations are regulated by the hypothalamus, whose role in the pathophysiology of BD has been discussed (48).

BD is associated with a high risk of SAs and deaths. It is estimated that the risk of suicide in BD patients is 20–30 times greater than in the general population (49). Epidemiological studies report that about 23%–26% of BD patients attempt suicide and that BD accounts for up to 14% of all deaths by suicide (50).

Intriguingly, in our clinical sample the sensitivity to weather changes was associated with the presence and number of lifetime SAs. This finding is relevant, since a history of a prior SA has a documented predictive role for completed suicide, and repeated attempts further increase the risk of death by suicide (51). There is evidence supporting the influence of seasonal changes on suicidal behavior that seems to be related to climatic variance and light exposure, possibly through an interaction with serotonergic and noradrenergic circuits (29, 47). Meteorological variables, such as temperature, atmospheric pressure, and sunlight, are linked to changes in the concentration of cerebral neurotransmitters and alterations in the serotonin turnover in the brain (47). A study by Makris et al. (52) found a correlation between sunshine and SAs in patients treated with serotonergic medications (i.e., SSRI). Therefore, environmental, weather-related neurobiological alterations of the serotonergic system might mediate suicidal behavior in predisposed individuals (47). Of note, dysfunctions in the serotonergic and dopaminergic systems have been also shown to play a role in the pathophysiological mechanisms underlying emotion dysregulation, which, in turn, is strongly linked to mood recurrences, impulsive behaviors, and substance abuse, ultimately leading to an increased risk of suicide in BD patients (53–55). Similarly, meteorological factors, including low barometric pressure, high temperatures and precipitations, have been associated with decreased sleep quality and abnormal circadian patterns (56, 57), which, in turn, may represent a potential marker of SA in BD (58).

Seasonal and climatic factors might interact with each other, influencing the manifestation of psychiatric symptoms mostly affecting suicide completers (59). Balestrieri et al. (60) suggested that the assessment of lifetime rhythmicity and manic-hypomanic features may be clinically useful to identify potential suicide attempters in high-risk groups. In a recent study, Bauer et al. (61) found an association between latitude-related changes in solar insolation and increased SAs in patients with BD. Finally, the weather has a significant impact on social activities and interactions, especially in vulnerable populations. Interpersonal stress, low social connectedness, and poor social support have been recognized as major risk factors for suicide (62–64).

In our study, women displayed a greater tendency to climatic sensitivity than men both in the clinical and in the control group, a finding in line with earlier observations (65). The greater influence exerted by seasonality and climatic changes on women could be determined by the different action of bioclimatic factors on neurohormonal systems.

Increased sensitivity to climate and weather variables, especially among fragile populations such as psychiatric patients, represents an issue to be considered in the light of the severe climatic modifications currently taking place. In fact, global climate change is now regarded as one of the biggest crisis facing humanity, and the impact of its consequences on mental health, both in adults and children, has been recognized as a major concern among mental health specialists (66–72). Accordingly, in 2017 the American Psychiatry Association (APA) released a position statement that climate change “poses a threat to public health, including mental health”, with people suffering from psychiatric disorders expected to be more dramatically impacted (73). This statement has been recently followed by an editorial of the European College of Neuropsychopharmacology (ECNP) President highlighting the need for more research on this field, and for further measures to mitigate the consequences of climate changes on mental health (74).

Increasing evidence indicates that climate change may affect mental health *via* multiple mechanisms, both direct and indirect, leading to outcomes ranging from psychological distress, fear, anxiety, or depression, to sleep disorders, substance use, and suicide (67, 70, 75). In our sample the symptomatic load, both physical and psychological, related to climate and weather variations was associated with lifetime SAs. Currently, suicide is one of the leading causes of death worldwide, and suicide rates in the U.S. have risen dramatically over the last 15 years, especially in adolescents and young adults aged 15 to 34 (76). Burke et al. described that temperature rising could increase the suicide rate by 1.4% in the U.S. and by 2.3% in Mexico, leading to an estimated number of about 21.000 additional suicides by 2050 in these countries (30). Therefore, a better understanding of the multiple causes influencing suicidal behaviors is indeed a public health priority.

Our results must be considered in the light of some issues referred to the design of the study, such as: the absence of objective measures of climatic parameters (i.e., latitude, barometric pressure, temperature, brightness, and precipitation); the reliability of a self-administered questionnaire (the METEO-Q), that may be partially biased as compared with semistructured interviews; the cross-sectional nature of the study, that does not follow individuals over time and does not help determine cause and effect. Therefore, our results in this sample may not be generalizable for other groups of subjects.

Our findings support the relevance of sensitivity to weather and climatic variations as a clinical characteristic in a large sample of BD patients. The association between sensitivity to weather changes and SAs could be useful in the assessment of suicide risk in BD patients.

Further studies that replicate our findings and investigate clinical and research implications are certainly needed.

## Data Availability Statement

The datasets analyzed for this study are available from the corresponding authors on reasonable request.

## Ethics Statement

The studies involving human participants were reviewed and approved by the Ethics Committee of the Fondazione Policlinico Universitario “A. Gemelli” IRCCS, Università Cattolica del Sacro Cuore, Rome, Italy. The patients/participants provided their written informed consent to participate in this study.

## Author Contributions

MN and LJ were primarily responsible for study design and contributed to data interpretation and article writing. MM, GC, and PG performed data collection and contributed to data interpretation. GG and GM were involved in data entry and database management. MN performed statistical analysis. MN, MM, MP, and DDB were involved in data interpretation. IP, LM, and GG contributed to data interpretation and article writing. All authors personally revised and approved the final version of the manuscript.

## Conflict of Interest

The authors declare that the research was conducted in the absence of any commercial or financial relationships that could be construed as a potential conflict of interest.
